# London

**Published:** 1914-09-05

**Authors:** 


### THE MEDICAL STUDENT AND HIS WORK.

### THE MEDICAL SCHOOLS OF THE UNITED KINGDOM.

### LONDON.

*St. Bartholomew's Hospital Medical School.*—St. Bartholomew's is the oldest and second largest hospital in London. The Medical School is complete in every department, and provides lectures and practical laboratory teaching in the preliminary sciences and intermediate subjects, as well as in the purely professional and clinical parts of the curriculum. Scholarships and prizes to the total value of £1,000 are awarded annually.

*Charing Cross Hospital.*—This hospital is fully organised for University teaching, and contains 225 beds and its Medical School. The courses of study are specially designed to meet the requirements of the University of London degrees, the Conjoint Board diplomas, and the final studies of the Universities of Oxford and Cambridge. Fully equipped laboratories are available for pathological, bacteriological, and public health work. Total fees: For general students—Sessional payments: Entrance fee 10 guineas and 25 guineas annually for University students, 22 guineas annually for Conjoint Board students, so long as the student remains in the school. Special arrangements are made to permit of fees being paid in advance. The usual resident and students' appointments are open to students, and several valuable prizes are awarded annually by the school. Full information respecting the Medical School, and the course of study recommended, may be obtained on application to the Dean, Dr. William Hunter, F.R.C.P., Medical College, Charing Cross Hospital, London, W.C.

*St. George's Hospital.*—The winter session commences on October 1, when A. E. Shipley, Esq., M.A., F.R.S., Master of Christ's College, Cambridge, will deliver the Inaugural Address, taking for his subject "Zoology and Medicine." Students can enter at any time. Special courses are given, in which the requirements of University and other examinations receive careful attention. The hospital contains 350 beds, and has a convalescent hospital of 100 beds at Wimbledon. The usual resident and students' appointments are open to students. Scholarships and endowed prizes, amounting to £463, are awarded every year. The entire teaching and laboratories are now devoted to purely clinical subjects, as in other Universities, to the great advantage of students in their fourth and fifth years of study. Arrangements have been made with the University of London for students who enter St. George's during the first, second, or third year of the curriculum to carry out the necessary courses of instruction at either University College or King's College. Students have therefore the advantages of the lectures and practical classes of these Colleges of the University during the preliminary and intermediate portions of their studies, and then complete their course in a school entirely devoted to medicine, surgery, and the study of disease. The St. George's Hospital Club has its athletic ground of 13½ acres on the Atkinson-Morley Hospital estate at Wimbledon. It provides a football and hockey ground and six tennis courts. Further particulars may be obtained on application to the Dean of the Medical School, Dr. R. Salusbury Trevor.

*Guy's Hospital.*—This hospital contains 618 beds, and has a large Medical School. There are numerous special departments, and a large number of student and resident appointments available. Special classes are held for the London University, Oxford, Cambridge, and Fellowship courses. Scholarships and prizes to the amount of £800 are awarded annually. For further particulars application should be made to the Dean of the Medical School, Dr. H. C. Cameron.

*King's College Hospital.*—This, the newest hospital in London, at present contains 280 beds, but will eventually be increased to the ultimate number of 600. It is situated at Denmark Hill, S.E., where an immense population is available for clinical work. Although the new hospital was opened so recently as last October, there are about 3,500 attendances per week in the casualty and out-patient departments. The hospital possesses every modern requirement for both patients and students. The teaching of the preliminary subjects, including chemistry, physics, biology, anatomy, and physiology, is given at King's College in the Strand, students coming to the hospital for their advanced work. Sixteen resident appointments are made every year. The composition fee for advanced medical studies is 70 guineas if paid in one instalment, and 72 guineas if paid in two instalments. The composition fee for the whole University or Conjoint course is 150 guineas. University Entrance Scholarships are competed for this month (September). Full particulars of these and the new prospectus may be obtained by applying to the Dean of the Hospital, or to the Secretary of the Medical School, Mr. Clifton Kelway.

*St. Mary's Hospital Medical School.*—St. Mary's Hospital contains at present 305 beds. Systematic clinical teaching is given daily in the wards and out-patients' department and in the various special departments of the hospital. The Medical School provides for the entire curriculum, and contains well-organised departments for preliminary scientific subjects. The composition fee for the whole curriculum is £140, or 60 guineas for the clinical curriculum only.

*The Middlesex Hospital.*—The hospital and Medical School are situated in Mortimer Street, and only a few minutes' walk from Goodge Street Station (Hampstead and Charing Cross Tube), Oxford Circus Stations (Bakerloo and Central London Tubes), and Portland Road Station (Metropolitan Railway).

The hospital contains 440 beds, including special wards for cancer cases, maternity and gynæcological cases, and for diseases of the skin and eye.

The cancer wing (containing ninety beds) and special investigation laboratories offer unrivalled opportunities for the study of cancer, both in its clinical and pathological aspects.

In the electro-therapeutical department students obtain instruction in the treatment of lupus and cancer by the *x*-ray method. An electro-cardiograph has recently been installed in this department.

The Bland-Sutton Institute of Pathology, which is just completed, contains a new lecture theatre, large laboratories for pathological, bacteriological, and clinical investigation, and smaller rooms for original research work. The Anatomical and Pathological Museum has been rebuilt and now forms part of the Institute.

There is a Residential College for a limited number of students.

Six house physicians, eight house surgeons, two obstetric and gynæcological surgeons, two casualty medical officers, two casualty surgical officers, and two resident officers to the special departments are appointed annually, without extra fee of any kind. Non-resident qualified clinical assistants are appointed to assist in the various out-patient departments.

Three Entrance Scholarships, value £100, £50, and £25, and a University Scholarship, value £50 (for Oxford and Cambridge students), are awarded annually in September. The successful candidates are required to become general students of the school. Numerous scholarships, medals, and prizes, to the value of over £1,000, are open for competition among students of the school.

All students join the Amalgamated Students' Club, which includes the following :—The Medical Society, the Common Room Society, the Cricket Club, the Football Clubs, the Athletic Clubs, the Rowing Club, the Swimming Club, the Musical Society, the Chess Club, the Lawn-tennis Club, and the Hockey Club. The athletic ground, which is eight acres in extent, is situated within easy access of the hospital—at Park Royal. There is a gymnasium within the precincts of the hospital.

The winter session, 1914-15, will open on Thursday, October 1, at 3 P.M. The Introductory Address will be delivered by C. Hubert Bond, Esq., M.D., D.Sc., M.R.C.P., after which the prizes will be distributed by H.S.H. Prince Alexander of Teck.

The annual dinner will be held on the same evening at seven o'clock at the Trocadero, Sir John Bland-Sutton, F.R.C.S., in the chair.

For prospectus and full particulars application should be made to the Dean of the Medical School.

*The London Hospital Medical College.*—The London Hospital is the largest institution of its kind in England, and contains 922 beds. Being situated in the East End, it ministers to a very large and poor district. The total number of patients during 1913 was 190,870, made up of 17,096 in-patients and 173,774 out-patients. During the year 6,751 major operations were performed in the theatre, while the number of operations under anæsthesia was 22,080. Five entrance scholarships are offered annually, including the Price Scholarship in science, of £100; the Epsom Scholarship; and the entrance scholarships in science, anatomy and physiology, and arts. The annual inclusive fee is 30 guineas. Appointments : Registrars, resident accoucheurs, house physicians, house surgeons, etc. One hundred and forty-one appointments are made annually. All appointments are free to full students, and all resident appointments have board free. Full information may be obtained from the Dean of the Medical College. A Hostel for students is provided on the hospital ground. The Clubs Union Athletic Ground is within easy reach of the hospital.

*London Hospital Dental School.*—The school, which is fully equipped on the most modern lines and with the latest appliances, is an integral part of the college and hospital, and is admirably adapted for the purpose of teaching. The school possesses, in addition to the theatres, laboratories, and museums of the college, a special museum of dental anatomy and surgery, operative dentistry, prosthetic and extraction rooms, and laboratories for practical dental metallurgy, dental prosthesis, and porcelain work. A systematic course of instruction in dental prosthesis is arranged for pupils. The up-to-date laboratory contains every modern apparatus, and is in charge of a skilled curator and his assistants. Students in the school will enjoy the great advantage of pursuing the medical portion of the dental curriculum in a well-equipped and successful college and hospital, of which the Dental School is part. Lecturers : Chemical and Dental Metallurgy, Hugh Candy; Physics, A. H. Fison; Human Anatomy, Professor W. Wright; Physiology, Professor L. Hill; Dental Surgery and Pathology, F. M. Farmer; Operating Dental Surgery, G. Northcroft; Operating Dental Prosthesis, G. P. Pollitt; Odonto-prosopic Orthopædics, H. Chapman; Dental Anatomy, Professor W. Wright; Dental Microscopy, E. Sprawson; Dental Bacteriology, Professor W. Bulloch; Dental Materia Medica, O. Grünbaum; Anæsthetics, R. J. Probyn-Williams.

*University College Hospital Medical School* comprises the departments of medicine and clinical medicine, surgery and clinical surgery, midwifery and gynæcology, pathology and morbid anatomy and clinical pathology, cardiography, bacteriology, mental physiology and mental diseases, dental surgery, practical pharmacy, and other departments for the study of special diseases, such as those of the eye, skin, ear, and throat, and for instruction in the use of anæsthetics, and in electro-therapeutics and the application of the $x$-rays. Scholarships, exhibitions, and prizes to the value of over £1,000 are offered for competition every year. Composition fees for the courses required by the University of London : For the final M.B., B.S. course, 80 guineas, or in two instalments of 50 and 32 guineas; for the medical education required by the Examining Board in England and the Society of Apothecaries, for the course required for the third examination, 80 guineas, or in two instalments of 50 and 32 guineas. The National Dental Hospital and School has recently been taken over as the Dental Department of University College Hospital. All students joining the Dental Department will do so as students of University College Hospital. The fee for the complete dental curriculum is £175, or, if paid by instalments, 50 guineas per annum for the first three years and 25 guineas for the fourth year. Qualified medical men wishing to obtain the special dental diploma of the Royal College of Surgeons can be admitted for the necessary two years' curriculum, including practical dental mechanics, operative work, and all the required dental lectures, for a fee of £120.

*University of London, University College* (Faculty of Medical Sciences) : University Centre for Preliminary and Intermediate Medical Studies.—The College Faculty of Medical Sciences comprises the departments of physics, chemistry, botany, and zoology (the preliminary medical sciences), also the departments of anatomy, physiology, and pharmacology (the intermediate medical sciences), and the departments of hygiene and public health, and pathological chemistry (post-graduate study). Composition Fees : For the courses required by the University of London : (1) For the first medical course, 26 guineas, entitling to one attendance. (2) For the second medical course, 58 guineas if paid in one sum; 60 guineas if paid in two instalments of 30 guineas each. This fee entitles to attendance on anatomy and physiology during three years, and to one attendance on organic and applied chemistry, pharmacology and materia medica, and to the privileges of the Union Society (including the use of the gymnasium and the athletic ground at Perivale) for two sessions. For the medical education required by the Examination Board in England and the Society of Apothecaries : First examination, Parts I., II., III., 21 guineas, entitling to one attendance and to the privileges of the Union Society for one session. First examination, Part IV., and second examination, 58 guineas, if paid in one sum, and 60 guineas if paid in two instalments of 30 guineas each. This fee entitles to attendance during three years (including one attendance on each practical course in physiology) in all subjects but practical pharmacy, the fee for which is three guineas, and to the privileges of the Union Society (including the use of the gymnasium and the athletic ground at Perivale) for two sessions.

*Westminster Hospital Medical School.*—Westminster Hospital was the first institution of its kind in London which was founded by the voluntary contributions of the public. There is accommodation for upwards of 200 in-patients. There is a Students' Clubs Union, the subscription for membership of which is included in the general fees. The annual composition fee is 25 guineas. Special terms are given to sons of medical men. Five Entrance Scholarships are offered for competition amongst students entering in October and two for those entering in May. Sessions begin: October 1, January 7, and April 22. Annual dinner, October 1, at Imperial Restaurant, Regent Street, W.

*Royal Hospital for Diseases of the Chest, City Road, E.C.*—This hospital, which has lately undergone extensive reorganisation, provides an excellent field for research and study in diseases of the chest, more especially in tuberculosis in its various departments. The departments in which study can be pursued comprise the in-patient, out-patient, Röntgen ray, cardiological, pathological and bacteriological, ear, nose and throat, and dental departments. An extensive department for the prevention of consumption (tuberculosis dispensary) has been established for nearly two years. As this works in connection with two of the large boroughs—namely, Shoreditch and Islington—a splendid opportunity presents itself for medical practitioners and students who desire to obtain an intimate knowledge of this work. The bacteriological and pathological research laboratories, under the new director, Dr. Carnegie Dickson, should provide excellent opportunities for acquiring a knowledge of bacteriological methods, and also a field for research for those who wish to pursue such studies. The cardiological department, which has recently been established, has been equipped with all the latest apparatus for the investigation of diseases of the heart. In connection with the Röntgen ray department, there are two radiologists, who attend the hospital four days weekly. Attached to the hospital is a Medical School for instruction in diseases of the chest, more especially tuberculosis, at which courses of instruction are given from time to time. There is also a tuberculosis school for nurses, where regular theoretical and thoroughly practical courses of instruction are given, for which a certificate is granted. The fees for attending the clinical practice of the hospital are as follows: One month, 1 guinea; three months, 3 guineas; six months, 5 guineas; perpetual ticket, 6 guineas. Any further information will be gladly given by the Dean, Dr. Barty King.

*London (Royal Free Hospital) School of Medicine for Women.*—The Royal Free Hospital is in Gray's Inn Road, and the London School of Medicine for Women is in Hunter Street, Brunswick Square, W.C. There are 165 beds in the hospital, all of which are available for clinical teaching. A new wing is about to be opened containing new and enlarged departments for out-patients, casualty, midwifery (11 beds), *x*-ray and electrical work, massage and dentistry. Also new and enlarged students' quarters. There are numerous scholarships and prizes offered annually in connection with the school, among which may be mentioned the Isabel Thorne Scholarship of £30, the St. Dunstan's Medical Exhibition of £60 a year for three years, extendible to five years, the Mabel Sharman-Crawford Scholarship of £20 a year for four years, and the Agnes Guthrie Bursary for dental students of £60. Various resident appointments at the Royal Free Hospital are available for students of the school after qualification. Five hospitals in London are entirely officered by medical women.

*The Hospital for Women, Soho.*—In connection with the out-patient department there has been for some years a well-organised clinical department. To meet the want increasingly felt by medical practitioners of a more intimate knowledge of the diseases peculiar to women, and of more extended opportunities for examining gynæcological cases, clinical assistants are appointed to the honorary medical officers to out-patients. The appointments are open to qualified medical men and women.

The hospital contains sixty-seven beds. In the out-patient department there are 4,000 new cases during the year, the total number of out-patient attendances being from 14,000 to 16,000. This large number affords exceptional opportunities for seeing and studying most of the varieties of the diseases of women. Clinical assistants are entitled to receive notice of all operations performed within the hospital upon giving their names and addresses to the hall porter, and every facility is afforded them by the honorary medical officers in the out-patient department of examining patients and obtaining experience in diagnosis and treatment.

The number of clinical assistants appointed to each gynæcologist is so far as possible limited to two, though occasionally a third may be appointed. The clinical assistants attend on two afternoons a week, and it is advisable that they should take out a three months' course, but to meet the convenience of medical practitioners it is permitted to attend for a shorter period, and on one or more days a week if it can be arranged.

A fee is charged at the rate of two guineas a month, which becomes due on joining. A certificate is given at the end of a three months' course.

The out-patients are seen daily at 1.30, except on Saturdays, when they are seen at 9.30.

Any further information can be obtained by writing to or calling on the Secretary at the hospital.

